# Calcification in Atherosclerotic Plaque Vulnerability: Friend or Foe?

**DOI:** 10.3389/fphys.2020.00056

**Published:** 2020-02-05

**Authors:** Xuan Shi, Jie Gao, Qiushi Lv, Haodi Cai, Fang Wang, Ruidong Ye, Xinfeng Liu

**Affiliations:** ^1^Department of Neurology, Jinling Hospital, Nanjing Medical University, Nanjing, China; ^2^Department of Neurology, Jinling Hospital, Southeast University, Nanjing, China; ^3^Department of Neurology, Jinling Hospital, Medical School of Nanjing University, Nanjing, China

**Keywords:** calcification, plaque, atherosclerosis, inflammation, optical coherence tomography, pathology

## Abstract

Calcification is a clinical marker of atherosclerosis. This review focuses on recent findings on the association between calcification and plaque vulnerability. Calcified plaques have traditionally been regarded as stable atheromas, those causing stenosis may be more stable than non-calcified plaques. With the advances in intravascular imaging technology, the detection of the calcification and its surrounding plaque components have evolved. Microcalcifications and spotty calcifications represent an active stage of vascular calcification correlated with inflammation, whereas the degree of plaque calcification is strongly inversely related to macrophage infiltration. Asymptomatic patients have a higher content of plaque calcification than that in symptomatic patients. The effect of calcification might be biphasic. Plaque rupture has been shown to correlate positively with the number of spotty calcifications, and inversely with the number of large calcifications. There may be certain stages of calcium deposition that may be more atherogenic. Moreover, superficial calcifications are independently associated with plaque rupture and intraplaque hemorrhage, which may be due to the concentrated and asymmetrical distribution of biological stress in plaques. Conclusively, calcification of differential amounts, sizes, shapes, and positions may play differential roles in plaque homeostasis. The surrounding environments around the calcification within plaques also have impacts on plaque homeostasis. The interactive effects of these important factors of calcifications and plaques still await further study.

## Introduction

There are three main types of vascular calcification: medial Mönckeberg arterial calcification, intimal calcification, and infantile calcification (Speer and Giachelli, 2004). Medial arterial calcification is often seen in aged, diabetes mellitus, and chronic renal failure patients (Otsuka et al., 2014). However, intimal calcification is more frequently associated with atherosclerosis and was regarded as a passive and degenerative process. It has been recognized as an active and self-regulated process (Amizuka et al., 2012). Intimal calcification is akin to the multistep process of bone formation and is controlled by complex enzymatic and cellular pathways (Speer and Giachelli, 2004). Vascular smooth muscle cells and other cell types (e.g., microvascular pericytes and adventitial myofibroblasts) have the potential to generate a mineralized matrix and undergo osteoblastic differentiation, then resulting in calcified deposits (Proudfoot et al., 1998; Reynolds et al., 2004; Demer and Tintut, 2008). Moreover, intimal calcification accelerates with bone-related proteins, such as bone morphogenetic protein-2 and -4, and osteocalcin (Zebboudj et al., 2002; Li et al., 2008; Panizo et al., 2009), and declines with calcification inhibitor proteins, such as osteopontin (Proudfoot et al., 1998; Scatena et al., 2007).

The role of calcification in atherosclerosis remains unclear. Several studies have revealed that the presence of calcification does not differ between symptomatic and asymptomatic arteries (de Weert et al., 2009; Jones et al., 2014). Calcified plaques causing stenosis may be more stable than non-calcified plaques (Nandalur et al., 2005). Conversely, severe stenotic (>70%) lesions have been reported to be linked with severe calcifications located on the outside layer of the atheroma (calcified burdens located away from the enhanced vascular lumen and/or outside the plaque) (Yamada et al., 2014). Moreover, calcified plaque is an independent predictor of combined vascular events (Prabhakaran et al., 2007; van den Bouwhuijsen et al., 2015) and recurrent stroke (Liu et al., 2012). Small calcifications may represent a dynamic inflammation-stimulated process, which has been associated with accelerated disease progression and greater atheroma burden (Abedin et al., 2004; Aikawa et al., 2007; Kataoka et al., 2012). In this review, we will focus on the effect of atherosclerotic calcification on plaque progression and vulnerability.

## Calcification Size and Plaque Vulnerability

### Microcalcifications

Microcalcifications (0.5–50 μm) have been found in atheromas (Vengrenyuk et al., 2008), and may represent an early stage in the continuum of the vascular calcification cascade (Reith et al., 2018). Plaque calcification reflects an active stage of atherosclerosis associated with inflammation. Inflammatory cytokines, such as tumor necrosis factor-α, activate osteogenic differentiation and mineralization of the extracellular matrix (Tintut et al., 2000). A stiffer matrix leads to the formation of calcific minerals (Kapustin et al., 2011; Hutcheson et al., 2016; Krohn et al., 2016; Roszkowska et al., 2018), provocation of inflammatory cytokines (New et al., 2013; Nakasaki et al., 2015), and lipid retention (van der Hoek et al., 1994). Then both macrophages and smooth muscle cells produce matrix vesicles (New et al., 2013; Kapustin et al., 2015), which may serve as initiation sites for mineral crystal formation, resulting in microcalcifications (Kapustin et al., 2015; Hutcheson et al., 2016). Microcalcifications coalesce into large masses, extend from the deeper region of the necrotic core into the surrounding collagenous matrix, and finally form calcified sheets or plates (Otsuka et al., 2014).

Microcalcification can be non-invasively identified by ^18^F-sodium fluoride (^18^F-NaF) positron emission tomography (PET)/CT imaging (Derlin et al., 2010; Dweck et al., 2012; Chen and Dilsizian, 2013; Joshi et al., 2014; Irkle et al., 2015; Vesey et al., 2017). PET-CT imaging with ^18^F-NaF can identify important features of plaque vulnerability by selectively detecting nascent and active microcalcifications. This is mainly because the extent of fluoride adsorption depends on the surface area of the mineral (Lin et al., 1981; Gasser et al., 1993). With the attenuation of X-rays, microcalcifications, which can be detected by ^18^F-NaF, might be absent on CT (Derlin et al., 2010; Hop et al., 2018). ^18^F-NaF activity was increased in areas without calcifications on CT that histology revealed to contain microcalcifications (Vesey et al., 2017; Hop et al., 2018). Most of the calcifications on CT, with high calcium scores, showed minimal ^18^F-NaF uptake (Fiz et al., 2015; Vesey et al., 2017; Hop et al., 2018). A preferential adsorption of ^18^F-NaF to microcalcifications with a relatively large surface area causes an intense signal on PET-CT images (Joshi et al., 2014; Irkle et al., 2015; Vesey et al., 2017). ^18^F-NaF is unable to penetrate through the deeper mineral layers in advanced macrocalcifications and binds only on the outer surface layer (Irkle et al., 2015). Moreover, PET-CT imaging with ^18^F-NaF potentially provides distinct information of high-risk pathology. ^18^F-NaF seems more closely associated with the process of necrotic inflammation and plaque metabolic activity (Joshi et al., 2014). Although the association between ^18^F-NaF positive plaques and ruptured or high-risk plaques has been demonstrated (Joshi et al., 2014; Oliveira-Santos et al., 2017; Vesey et al., 2017), the role of microcalcification itself in high-risk plaques or vulnerable patients has not been further explained.

**FIGURE 1:**
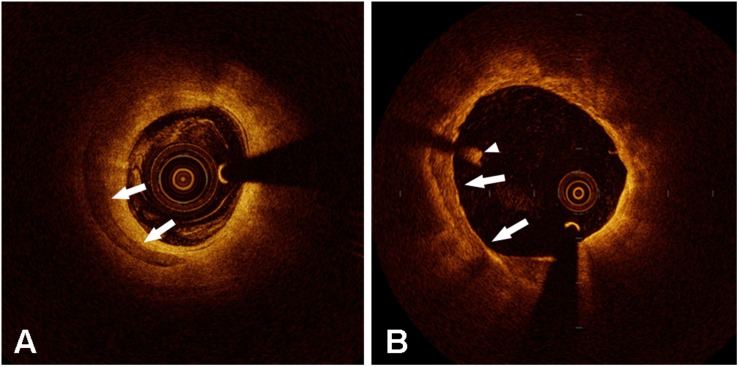
Representative optical coherence tomography images of calcification (arrow in panels **A** and **B**). The arrowhead shows an intraluminal white thrombus.

Few optical coherence tomography (OCT) studies have attempted to investigate the effects of microcalcification on plaques. OCT has the highest resolution with 10–15 μm axially and 20–40 μm horizontally of any intravascular imaging modality. OCT can help us evaluate more detailed plaque morphologies and calcification characteristics (Figure 1). Although OCT still has difficulties in visualizing cellular-level microcalcifications, it provides the potential for investigating the clinical and morphological predictors of microcalcifications *in vivo*. OCT-defined microcalcifications are the calcium deposits with a maximum calcium angle <22.5° and a maximal calcification length <1 mm (Milzi et al., 2017). OCT-defined microcalcifications have been demonstrated to be related to milder stenosis with extensive plaque inflammation (Reith et al., 2018) and a large necrotic core (Kim et al., 2016). In plaques with microcalcifications, a higher rate of macrophage infiltration was demonstrated by measuring various parameters including macrophage angle, length, and volume index (Reith et al., 2018). OCT-defined microcalcifications may represent an active, early stage of vascular calcification (Reith et al., 2018), suggesting they might be at the same stage as cellular microcalcifications. However, in Reith’s study, the inclusion of patients with stable coronary heart disease and the exclusion of patients with acute coronary syndrome (ACS) limited this conclusion to some degree. These regions where microcalcification and macrophage infiltration co-localize might not be all the same as plaques leading to ACS.

The main mechanism of ACS is plaque rupture and subsequent thrombosis. Cellular-level microcalcifications might be one of the important causes of plaque stress concentration and rupture. Microcalcifications are found in nearly all fibrous caps (Kelly-Arnold et al., 2013) instead of being a relatively rare occurrence (Vengrenyuk et al., 2006). Microcalcifications are more likely to be heterogeneous, but only a small subset have the potential for rupture (Kelly-Arnold et al., 2013). Few studies have suggested that microcalcifications may not be detrimental *per se* unless they are located in a region of high background stress (Vengrenyuk et al., 2008), such as thin fibrous caps or cap shoulders (Vengrenyuk et al., 2006; Cardoso et al., 2014). Microcalcifications might cause the transfer of regional high stress. The stress concentration moves from the interface between the fibrous cap and lipid core to the interface between the fibrous cap and vessel lumen (Rambhia et al., 2012), leading to plaque rupture. However, other studies suggested that microcalcifications can increase the local stress independent of the size of the particle and are relatively insensitive to its position in the fibrous cap (Kelly-Arnold et al., 2013). If microcalcifications are located in regions where background stress has been elevated, the increased stress due to microcalcifications might not be substantial enough to cause rupture, especially in the region where the cap is thin (Kelly-Arnold et al., 2013). Nonetheless, when two microcalcifications are located in close proximity (the gap between microcalcifications/their diameters < 0.4), the stress concentration factor rises exponentially, and the whole region is exposed to high stress (Kelly-Arnold et al., 2013). Only when this considerable stress increase is in a location where background stress is nearly sufficient to rupture could cap rupture happen. Based on these findings, small particles such as microcalcifications < 5 μm are not harmful because of their insufficient force caused by the tiny voids (Kelly-Arnold et al., 2013).

### Spotty Calcifications

Spotty calcifications were initially defined as small calcium deposits within an arc of <90° with intravascular ultrasound (IVUS) by Yoshikawa’s group (Ehara et al., 2004). This definition was then applied in IVUS (Fujii et al., 2005) and OCT (Sakaguchi et al., 2016) studies. The arc of calcification is defined as the widest angle in which the calcifications were detected and measured in each IVUS or OCT image slice (Fujii et al., 2005; Milzi et al., 2017). For CT, spotty calcifications were defined when calcifications <3 mm in size were observed on curved multiplanar reformation images (Motoyama et al., 2007). Yoshikawa’s group demonstrated that the length of the calcification exhibited a positive correlation with the largest arc (Ehara et al., 2007). Larger and longer calcifications are associated with stable angina pectoris (SAP), whereas small calcifications are more common in patients with ACS (Ehara et al., 2007). Spotty calcifications have therefore been further illustrated to have an arc of <90° and calcification length of <4 mm in IVUS (Kataoka et al., 2012) and OCT analyses (Kataoka et al., 2014; Ong et al., 2016).

Spotty calcifications are associated with more extensive and diffuse atherosclerosis and accelerated disease progression (Kataoka et al., 2012). Kataoka et al. (2012) selected 1347 stable patients with angiographic coronary disease from seven prospective atherosclerosis progression and/or regression IVUS trials. Spotty calcifications were observed in 27% of stable patients, and these lesions had larger percentage atheroma volumes and total atheroma volumes with greater progression of atheroma volume percentages. Plaques with spotty calcifications were more often in fibroatheromas than in fibrocalcific plaques, and the median arc of calcium was 52° with smaller arcs in the setting of fibroatheromas than in fibrocalcific plaques in the histopathologic study (Pu et al., 2014). Moreover, spotty calcifications were more frequently found in ruptured plaques (Sakaguchi et al., 2016). Most spotty calcifications in ruptured plaques tended to be found in more shallow locations (Sakaguchi et al., 2016), which is more likely to contribute to rupture (Li et al., 2007; Zhongzhao et al., 2014).

However, even when the accelerated plaque does rupture due to spotty calcification, does it truly cause thrombolytic events? Spotty calcified lesions are observed more often in ACS than in SAP (Ehara et al., 2004; Mizukoshi et al., 2013). The culprit segments in acute myocardial infarction (AMI) patients were mostly characterized by spotty calcifications (Ehara et al., 2004). The significance of spotty calcifications in the pathogenesis of ACS was recently questioned (Ong et al., 2016). Given that the culprit lesions of ruptured plaques in ACS are 16 mm in length on average (Jia et al., 2013), calcifications remote from culprit rupture sites are unlikely to cause cap rupture. There was a trend toward a higher number of spotty calcifications in ruptured plaques with ST-segment-elevation myocardial infarction than plaques with SAP when performing a full 30 mm segment analysis, whereas no significant difference in spotty calcifications was found with a 10 mm segment analysis (Ong et al., 2016). The culprit lesion in these studies was selected using the same criteria as those used for the image slice with the smallest minimum luminal area on cross-sectional imaging. Nonetheless, Ehara et al.’s (2004) study also focused calcium analysis on 10-mm-long culprit lesion segments. Spotty calcifications responsible for ACS might not be equivalent to spotty calcifications causing plaque rupture. The environment surrounding the spotty calcification within plaques could play an important role in plaque homeostasis. Disruptive plaque homeostasis will contribute to clinical events, and spotty calcification may act as a fuse. This hypothesis could be supported by the fact that the benefits of LDL-lowing therapies on atheroma progression are significantly attenuated in patients with spotty calcification (Kataoka et al., 2012). Intensive statin therapy has a stronger effects on patients with spotty calcification than does moderate statin therapy (Afolabi et al., 2018).

**FIGURE 2:**
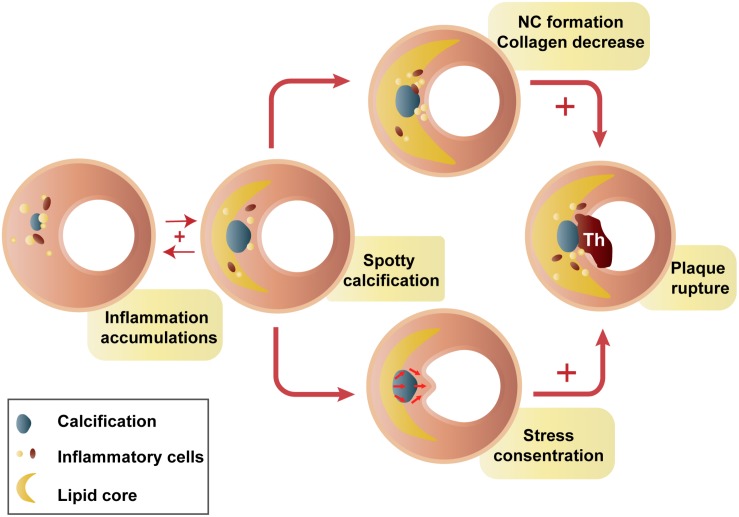
Hypothesis of spotty calcification in plaque rupture. Two main ways that spotty calcifications lead to plaque rupture: inflammatory cytokines from macrophages activate osteogenic differentiation, contributing to early stages of calcification. Then, a positive feedback loop between inflammation and calcification produces spotty calcification, stimulating accelerated plaque progression, including greater inflammatory burden, a larger necrotic core, and less collagen (Aikawa et al., 2007; Kataoka et al., 2012; Pu et al., 2014, 2016a; Hsu et al., 2016). In contrast, calcification neighboring the lipid pool, especially that near the fibrous cap, may intensify failure stress and cause plaque rupture (Bluestein et al., 2008; Hoshino et al., 2009). NC, necrotic core; Th, thrombus.

Spotty calcifications might have impacts on plaque homeostasis through inflammation immersion. Spotty calcification, but not extensive calcification, is correlated with greater inflammatory burden, proinflammatory factor expression (Pu et al., 2016a), and collagen synthesis reduction (Pu et al., 2016b). Macrophage-derived cytokines result in osteogenic differentiation and mineralization of vascular smooth muscle cells (Radcliff et al., 2005) and then generate atherosclerosis-associated small calcifications (Aikawa et al., 2007). Conversely, a strong inverse relationship between the degree of plaque calcification and macrophage infiltration was found in both symptomatic (*r* = −0.78) and asymptomatic (*r* = −0.89) plaques (Shaalan et al., 2004). It seems to be a positive feedback amplification loop between small calcifications and inflammation. Small calcifications may provoke additional proinflammatory responses (Bostrom, 2005; Nadra et al., 2005), thereby stimulating plaque calcification and disease progression. Small calcifications, especially those located near the lumen or lipid core, could intensify circumferential stress and cause plaque rupture (Li et al., 2007; Hoshino et al., 2009; Zhongzhao et al., 2014). This may provide a possible explanation for why spotty calcifications are positively related to plaque rupture (Figure 2; Kataoka et al., 2014; Sakaguchi et al., 2016). There are certain stages of calcium deposition that may be more atherogenic and vulnerable (Bostrom, 2005). In contrast, end-stage calcifications, traditionally viewed as irreversible, are more likely to play a role in increasing tissue mineralization and limiting inflammation (New and Aikawa, 2011).

### Large Calcification

There has been no clear definition of large calcification. With traditional imaging methods such as computed tomography angiography (CTA) or magnetic resonance angiography (MRA), most studies evaluated calcium content, including calcification volume, calcification area, and percent area calcification for each plaque. Plaque calcification was higher in asymptomatic patients than in symptomatic patients (Miralles et al., 2006). Additionally, the percent plaque area calcification was twofold greater in asymptomatic than in symptomatic plaques. In contrast, Magge et al. (2013) found that calcification volume was larger in patients with stroke on the infarct side than in those with stroke on the non-infarct side and the patients with no stroke. Furthermore, a larger calcification volume is associated with a higher prevalence of intraplaque hemorrhage (IPH) (van den Bouwhuijsen et al., 2015). These contradictory conclusions might be because all of these comparisons in calcification size were qualitative in each study.

For IVUS or OCT, calcification arc and length have been employed to distinguish spotty calcification and large calcification. A moderate calcific lesion with an arc of 90° to 180° in >1 cross-sectional image of the lesion was defined as intermediate calcification, and a calcific lesion with an arc of >180° in >1 cross-sectional image was defined as extensive calcification with IVUS (Ehara et al., 2004). Large calcifications were classified as calcium deposits with an arc of >90° with OCT (Mizukoshi et al., 2013; Ong et al., 2016; Sakaguchi et al., 2016). Such large calcifications were found to correlate inversely with plaque rupture (Mizukoshi et al., 2013). Extensive calcification, with an arc of >180°, was the most frequent (38%) type in SAP patients (Ehara et al., 2004). Other studies demonstrated no difference in large calcifications between ruptured and non-ruptured plaques (Ong et al., 2016; Sakaguchi et al., 2016). Thus, using this definition, large calcifications might be more strongly associated with stable plaques.

Given the different findings for spotty calcifications and large calcifications, the role of calcification size in plaque vulnerability might be biphasic (Abedin et al., 2004; Hsu et al., 2016). Failure stress is expected to be concentrated at the interfaces between materials of different stiffness. Within a plaque, mechanical stress concentrates at interfaces between calcium deposits and other vascular tissue (Buffinton and Ebenstein, 2014). The stiffness of calcium is at least four times greater than that of other plaque components (Lee et al., 1993). As the degree of calcification increases, the surface area increases until calcium deposits coalesce. However, calcium deposits continue to form and grow, and the surface area decreases afterward (Abedin et al., 2004). Nevertheless, this hypothesis is difficult to prove since the interface area of calcification is hard to observe and measure. Katano et al. (2015) divided calcification formation into two sections at a calcium score of 500. The symptomatic rate increases along with the calcium score until 500. Afterward, the symptomatic rate decreases with the calcium score archiving its highest level (>800) (Katano et al., 2015). This finding might be generally consistent with the hypothesis, but it does not give a good explanation of the core problem – the surface area. A promising novel biomarker, ^18^F-sodium fluoride, can reflect calcification activity. ^18^F-fluoride PET images make it possible to identify exposed hydroxyapatite crystals at the surface area of calcifications. Inversely, the internal area of calcifications, of which the hydroxyapatite core is internalized, may not be identified (Dweck et al., 2014). Therefore, PET-CT imaging with ^18^F-NaF might provide the potential for verifying whether the surface area of the calcification has a key effect on calcific plaque vulnerability.

## Calcification Number and Plaque Vulnerability

The calcification number has seldom been quantitatively explored. Table 1 shows recent studies on the relationship between calcification number and plaque vulnerability. Magge et al. (2013) found that the number of calcium clusters was similar between new carotid infarct and no-new carotid infarct patients by CTA. Calcium clusters were those with 20 or more “calcium” voxels adjacent to each other (Magge et al., 2013). This means that the number of calcium clusters does not exactly reflect the number of calcifications. With higher resolution imaging modalities, the results became different. Two high-resolution MRI studies classified plaques into single or multiple calcifications. Multiple calcifications have been observed more often in plaques with IPH and ulceration than in those with neither IPH nor ulceration (Yang et al., 2018). Of 142 carotid plaques in 117 patients with cerebrovascular symptoms, plaques with IPH showed a greater prevalence of calcification. After adjusting for age, low-density lipoprotein, maximum wall thickness, and maximum soft plaque thickness, multiple calcifications were strongly associated with IPH (Lin et al., 2017). Compared with single calcifications, multiple calcifications are more vulnerable and more common in the accelerated plaques together with ulceration or IPH. In addition, multiple calcifications would may provoke more activated inflammatory responses (Nadra et al., 2005), and then increase the probability of plaque rupture (Mizukoshi et al., 2013; Lin et al., 2017). On the other hand, the surface area mentioned above may still play an important role. The surface area will increase when the number of calcifications increases and the distribution of biomechanical stress in plaques becomes more complex.

**TABLE 1:** Studies on relationship between calcification number and plaque stability.

Authors	Year	Patients (Lesions)	Imaging	Vascular	Main results of the number of calcification
Ehara et al., 2004	2004	178 (178)	IVUS	Coronary	Average number of calcium deposits within an arc of <90°: AMI 1.4 ± 1.3, UAP 1.0 ± 1.1, SAP 0.5 ± 0.8, *p* < 0.0005
Fujii et al., 2005	2005	99 (106)	IVUS	Coronary	Ruptured plaques 3.5 ± 1.7, control plaques 1.8 ± 1.1, *p* < 0.001 At the section with minimum lumen CSA: ruptured plaques 53%, control plaques 65%, *p* = 0.1 Somewhere else than at the section with minimum lumen CSA: ruptured plaques 84%, control plaques 45%, *p* < 0.001
Mizukoshi et al., 2013	2013	187 (187)	OCT	Coronary	Average number of calcium deposits within an arc of <90°: AMI 2.0 (1.0–3.0), UAP 2.0 (1.0–3.0), SAP 1.0 (0.0–2.0), *p* < 0.001 Average number of calcium deposits within an arc of >90°: AMI 0 (0–1.0), UAP 0 (0–1.0), SAP 1.0 (1.0–3.0), *p* < 0.001
Ong et al., 2016	2016	108 (108)	OCT	Coronary	Total calcium deposits: STEMI-PR 1 (0–2.5), SAP 1 (0–3), *p* = 0.58 Spotty calcium (maximum arc <90° and length <4 mm): STEMI-PR 1 (0–2), SAP 1 (0–2), *p* = 0.87 Large calcium: STEMI-PR 0 (0–1), SAP 0 (0–1), *p* = 0.27
Sakaguchi et al., 2016	2016	98 (98)	OCT	Coronary	Spotty calcification (maximum arc <90°): rupture 79.0%, non-rupture 50.0%, *p* = 0.006 Number of spotty calcification per patients: rupture 1.5 ± 1.0, non-rupture 0.8 ± 0.9, *p* < 0.001 Large calcification: rupture 50.0%, non-rupture 53.3%, *p* = 0.84
Lin et al., 2017	2017	85 (145)	hrMRI	Carotid	Single calcification: with IPH 15%, without IPH 29.4%, *p* = 0.532 Multiple calcification: with IPH 72.5%, without IPH 26.5%, *p* < 0.001
Reith et al., 2018	2018	98 (112)	OCT	Coronary	Total calcifications: in segments with microcalcifications (maximum arc < 22.5° and length ≤ 1 mm) 6.7 ± 3.0, without microcalcifications 3.2 ± 2.5, *p* < 0.001 Number of calcifications independently predict the presence of microcalcifications: OR 1.53 (1.23–1.91), *p* < 0.001
Yang et al., 2018	2018	137 (137)	CTA, CTP	Carotid	Single calcification: ulceration (+) 26.5%, ulceration (−) 42.6%, *p* = 0.050; IPH (+) 28.2%, IPH (−) 44.1%, *p* = 0.053 Multiple calcification: ulceration (+) 47.0%, ulceration (−) 20.4%, *p* = 0.002; IPH (+) 52.1%, IPH (−) 19.7%, *p* < 0.001 Multiple calcification independently predict the presence of IPH: OR 3.9 (1.4–10.9), *p* = 0.009

*IVUS, intravascular ultrasound; OCT, optical coherence tomography; hrMRI, high resolution magnetic resonance imaging; CTA, CT angiology; CTP, CT perfusion; CSA, cross-sectional area; AMI, acute myocardial infarction; UAP, unstable angina pectoris; SAP, stable angina pectoris; STEMI, ST-segment-elevation myocardial infarction; PR, plaque rupture; IPH, intraplaque hemorrhage.*

Plaque rupture has been observed to correlate positively with the number of spotty calcifications (Sakaguchi et al., 2016) and inversely with the number of large calcifications (Mizukoshi et al., 2013). In addition, the number of spotty calcifications was significantly greater in AMI and unstable angina pectoris (UAP) patients than in SAP patients (Ehara et al., 2004; Mizukoshi et al., 2013). Thus, the effect of the number and area of calcifications on plaque stability cannot be discussed separately. Multiple small calcifications may increase the surface area, leading to higher stress concentrations and more unbalanced stress distributions. As such small calcifications grow and collapse, a large calcification will form, and the surface area will decrease (Abedin et al., 2004; Hsu et al., 2016). The calcium score might be the method that takes both the number and size of calcifications into consideration. The calcium score is calculated by multiplying the lesion area by a density factor and finally summing it for all individual lesions (Youssef et al., 2013). However, the results assessed by calcium scores are still contradictory. A positive relationship between a high calcium score and vascular events was investigated (Yang et al., 2012). However, a low calcium score was found to be an independent predictor for recurrent stenosis 1 year after carotid endarterectomy (CEA) or later on 94 calcified carotid plaques (Katano et al., 2017). The authors speculated that calcification may act as a physical barrier protecting against injury during CEA or inhibit myointimal hyperplasia. Nonetheless, this method treats all calcifications as a whole. One or two large calcifications and many small calcifications may achieve the same high calcium score and are included the same way in the analysis. Moreover, the effect of individual calcification on the plaque could not be investigated. The rupture of the fibrous cap may be induced by some high-risk calcifications.

## Calcification Location and Plaque Vulnerability

Calcifications in different locations of the vascular wall may play different roles in the plaque vulnerability. Several investigations have described the association between calcification location and vulnerable plaques. Superficial calcifications have been demonstrated to be related to plaque vulnerability. Nevertheless, the determination of superficial calcifications has not been unified. Superficial calcification was first introduced into the coronary artery by means of IVUS as calcification located at the intimal–luminal interface or closer to the lumen than to the adventitia. Deep calcifications were those at the media/adventitia border or closer to the adventitia than to the lumen (Mintz et al., 1995; Fujii et al., 2005). Xu et al. (2010) reported that marginal superficial calcification detected by high resolution MR showed a higher risk for the accompaniment of IPH. Two recent studies on carotid atherosclerotic plaque employed this definition to examine the association of calcification location and IPH. One study used carotid CTA source images to illustrate superficial calcifications as calcified nodules located at the intimal–luminal interface or close to the lumen (Yang et al., 2018). The other defined surface calcifications as calcified nodules within or very close to the fibrous cap but without complete coverage of fibrous tissue by means of MRI (Lin et al., 2017). For the OCT imaging, Jang’s group utilized 65 or 100 μm criteria to define superficial calcium and found no connection between calcium location and symptomatic plaques (Ong et al., 2016). However, their recent investigation demonstrated that sheet-like superficial calcific plates without erupted nodules or protruding masses into the lumen accounted for 67.4% of calcified plaques at the culprit site (Sugiyama et al., 2019). Yu’s group found that a minimum depth of 63 μm or even less of calcification is the critical cutoff point for coronary lipid-rich calcified plaque rupture (sensitivity = 77.8%, specificity = 81.8%) (Zhan et al., 2017).

Although the definition of superficial calcification varies, the association between superficial calcification and plaque vulnerability has been demonstrated (Xu et al., 2010; Lin et al., 2017; Zhan et al., 2017; Yang et al., 2018). During the complex physiological and biomechanical processes of IPH and plaque rupture, superficial calcifications could increase the local stress and stretch concentration (Zhongzhao et al., 2014). Structural analysis on idealized plaque models showed that calcification within thin fibrous caps may result in high stress concentrations and lead to plaque rupture (Li et al., 2007). Stiff calcium causes adverse stress within the plaque. The thinner the plaque cap is, the higher the stress concentrations it produces (Zhongzhao et al., 2014). A previous *ex vivo* study on biomechanical stability came to a different conclusion, suggesting that calcification does not increase fibrous cap stress in typical ruptured or stable human coronary atherosclerotic plaques (Huang et al., 2001). The reason might be that histological fixation might lead to the distortion of the specimen and may have an impact on the predicted stress contribution (Li et al., 2007). On the other hand, deep calcifications located away from the lumen might have little or no impact on plaque stress. Additionally, they could act as a barrier to the growth of vasa vasorum or the spread of inflammatory stimuli (Gossl et al., 2010), thereby reducing IPH or plaque rupture.

## Calcification Shape and Plaque Vulnerability

Several studies have tried to investigate calcification morphology including quantification of calcium shape score (Katano et al., 2015, 2017). Referring to the degree of each calcification encircling the vessel with CTA, the shape of the calcification was stratified and scored from 1 to 5: 1, less than one-quarter; 2, one-quarter to one-half; 3, one-half to three-quarters; 4, three-quarters to full circle; and 5, full circle covering the entire carotid perimeter (Katano et al., 2015). Low symptomatic calcified carotid plaques (<40%) tend to have calcification with significantly high calcium scores and high calcium shape scores (circularities) (Katano et al., 2015). Low circularity of calcification has a tendency to show more than moderate restenosis (≥50%) at 1 year after CEA (Katano et al., 2017). Conversely, increasing OCT-detected calcification also identifies patients at increasing risk for 1-year rates of myocardial infarction or death. Similar to the CT calcium shape score, patients were categorized according to the degree of calcium by OCT: none (calcium arc = 0°), mild (calcium arc = 1–180°), and severe (calcium arc = 181–360°) calcification (Singbal et al., 2016). Patients with mild and severe calcification were older with more frequent renal dysfunction, and had longer lesion lengths than those without calcification (Singbal et al., 2016). Both calcium shape scores reflect the circularities of calcium deposits in each plaque essentially, which are inextricably linked to the size of calcium deposits in plaque.

The potential effects of calcification particular shape on plaque vulnerability have been further investigated. In a high resolution MR analysis of 63 patients with carotid stenosis, irregular calcification is more frequently accompanied by IPH compared to patchy type (Xu et al., 2010). The item *irregular* in their study was defined as dotted or arcuated calcification. The patchy type was calcification, which occupied >50% of the plaque. Another MR study found that thin calcification (the maximum thickness < 2 mm) was associated with IPH (Yang et al., 2018). Due to the limited resolution of imaging techniques, more detailed shape features of calcifications have not been analyzed *in vivo*. A computational study using idealized plaque models indicated that calcification shape may affect plaque stress (Buffinton and Ebenstein, 2014). The shape index defined as the calcification length/thickness was introduced to quantify calcification geometric parameters. The peak stress was more sensitive to thickness than arc, and more strongly associated with shape index than calcification area. In this study, the small spherical calcification adjacent to the lumen resulted in a 33% increase in stress in the fibrous tissue at the shoulders. This might be similar to the calcified nodules near the lumen, which has been identified as a vulnerable calcified morphology (Buffinton and Ebenstein, 2014). Another vulnerable morphology is arc-shaped calcification (Buffinton and Ebenstein, 2014), which may correlate with silent ruptures in calcified plaques (Mauriello et al., 2011).

The effects of calcification shape were further examined in a patient-specific computational study of 92 human coronary arteries (Kelly-Arnold et al., 2013). Using CTAn software after reconstructed high-resolution microcomputed tomography images from specimens, the volume and surface of each particle were measured and the approximate aspect ratio major axis/tensile axis was calculated. Elongated particles would cause a 3.7- and 4.2-fold increase in local stress. However, when its volume-equivalent sphere was calculated, local stress had only a twofold increase (Kelly-Arnold et al., 2013). A similar finding has been reported in that the local maximum stress concentration around the near spheroid calcification increased twofold, whereas it increased fourfold around the elliptical calcification (aspect ratio >2) (Vengrenyuk et al., 2008). Clearly, these cellular-level microcalcifications and the above calcification in MRA or OCT reflect different stages of calcification. The shape of calcification in the occurrence and development of calcification must be constantly changing. We cannot confirm the association between cellular-level microcalcification and macroscopic calcification evaluated by CT/MRI. Spheroid microcalcification may either develop into dotted calcifications or accumulate into arc-shaped calcifications in MRI. These studies sought to explore the relative contribution of calcification shape in certain stages to certain stages of plaques.

## Calcified Nodules in Plaques

Pathologically, calcified nodules show an underlying fracture of calcified plates with a disrupted fibrous cap and overlying luminal thrombus (Virmani et al., 2006). Calcified nodules should not be confused with fibrocalcific lesions, which appear to be the end result of fibrosis and are not associated with thrombi (Virmani et al., 2000). The precise mechanism underlying calcified nodules is unknown. One hypothesis is that calcium sheets or plates break and form small nodules surrounded by fibrin and then protrude into the lumen. When calcified nodules are produced in eccentric lesions, they are more likely to cause disruption of the overlying luminal endothelium and result in platelet adherence (Yahagi et al., 2016).

The frequency and distribution of calcified nodules have been assessed *in vivo* using intravascular imaging techniques. In the first clinical case report, three patients were misdiagnosed with intravascular thrombus by coronary angiography, but IVUS showed that the target lesions were calcified masses instead of thrombi (Duissailant et al., 1996). It is possible to use IVUS to classify atherosclerotic lesions referring to the American Heart Association (AHA) recommendations that were based on histological examination (Erbel et al., 1999). IVUS criteria for calcified nodules were subsequently established: (1) convex luminal surface (sensitivity 94.1%, specificity 90.3%), (2) a convex shape of the luminal side of calcium, (3) an irregular, non-smooth luminal surface (sensitivity 64.7%, specificity 88.4%), and (4) an irregular leading edge of calcium (Lee et al., 2011). The most important IVUS characteristic of a calcified nodule was the convex plaque shape (Lee et al., 2011). Compared with IVUS, OCT can visualize the microstructure of atherosclerotic plaques and has the potential to characterize calcification, particularly calcified nodules. In 2013, Jia et al. (2013) established the OCT criteria of OCT-detected calcified nodules: fibrous cap disruption detected over a calcified plaque characterized by a protruding calcification, superficial calcium, and the presence of substantive calcium proximal and/or distal to the lesion.

Calcified nodules often occur in severely calcified arteries. In an OCT study with 889 *de novo* culprit lesions, calcified nodules were found in 4.2% of all lesions. Lesions containing calcified nodules have a larger calcium arc, a longer calcium length, thicker calcium, and a more superficial location of calcium compared with those without calcium nodules (Lee et al., 2017). Moreover, calcified nodules in patients with ACS have more thrombi than those in patients with SAP (Lee et al., 2017). These findings are consistent with another OCT study (Kobayashi et al., 2018). Eruptive calcified nodules (expulsion of small calcific nodules into the lumen) showed predominantly red thrombi, whereas superficial calcific sheets had predominant white thrombi (Sugiyama et al., 2019). This result might be due to eruption of calcified nodules causing eruption of the endothelium and contributing to thrombus formation (Virmani et al., 2006). However, the sequence between the formation of calcific nodules and thrombosis cannot be clearly confirmed. Calcific nodules in the untreated non-culprit lesions with ACS seem to be benign, with fewer major adverse events during the 3-year follow-up (Xu et al., 2012). Plaques with calcific nodules in non-culprit lesions were more often thick-cap fibroatheromas (Xu et al., 2012), which has not been associated with increased events (Takaya et al., 2006; Narula et al., 2013). Nonetheless, calcific nodules at the culprit lesions were not evaluated in this study. There is still a possibility that the role of calcific nodules is associated with the distance it intrudes from the lumen. Calcific nodules that intrude into luminal surfaces when the distance from the estimated normal lumen to the actual lumen is ≥100 μm might be more vulnerable (Matsumoto et al., 2012). Therefore, whether calcific nodules are the end result of plaque rupture and thrombosis or are causative of events remains uncertain.

Calcified nodules might be an early and late cause of in-stent failure. Lesions with calcified nodules have smaller preintervention minimum lumen areas and reference lumen areas, as well as the postintervention minimum lumen cross-sectional area (Lee et al., 2017; Kobayashi et al., 2018). A smaller postintervention minimum lumen cross-sectional area and reference lumen area are predictors of revascularization and adverse cardiac events (Sonoda et al., 2004; Doi et al., 2009; Prati et al., 2015). These may account for a higher incidence of revascularization at 500 days in lesions with calcified nodules (Kobayashi et al., 2018). Moreover, even after stenting, the formation and protrusion of nodular calcification within the neointima or around the stent struts could lead to in-stent thrombosis and ultimately contribute to chronic occlusion (Mori et al., 2016). Thus, the management of calcified plaques in patients with ACS is still challenging. Lesion preparation involving fracture-resistant stents with constant hinge movement, rotational and orbital atherectomy could be taken into consideration.

## Calcification and Vascular Remodeling

Calcification may be associated with remodeling independent of inflammation (Burke et al., 2002). Both forms of calcium, either plates of calcium in fibrous plaque or granules of calcium in cores, are related to vascular remodeling (Burke et al., 2002). Calcifications morphologic features seem to be associated with vascular remodeling. In a preinterventional quantitative IVUS analysis of 178 patients (Ehara et al., 2004), spotty calcification was the most frequent pattern in AMI (67%) and UAP (47%) patients with positive remodeling. Vascular positive remodeling, which was first described as a compensatory enlargement, might delay the progression of luminal narrowing in the early stage of atherosclerosis (Glagov et al., 1987; Hermiller et al., 1993). However, it is more frequent in a complex, vulnerable lesion, which is characterized by hemorrhage, large lipid cores, infiltrates of macrophages, and calcium deposits (Nakamura et al., 2001; Burke et al., 2002; Yonetsu et al., 2016). This was consistent with spotty calcification within a fibrofatty plaque being the most frequent pattern in AMI and UAP patients with positive remodeling (AMI 79%, UAP 55%) (Ehara et al., 2004), suggesting that fibrofatty plaques with spotty calcification are more vulnerable to ACS.

Conversely, the frequency of extensive calcification is the highest in SAP patients with positive remodeling (44%) and negative remodeling (41%) (Ehara et al., 2004). This finding was consistent with previous IVUS data suggesting that fibrocalcific plaques were associated with negative remodeling (Tauth et al., 1997). When the lumen decreases, calcific material might lead to a retraction of the lesion in response to increasing wall shear stress (Stiel et al., 1989). Instead, lipids might be more prone to enlargement (Lafont et al., 1995). Thus, lesions with a large arc of calcium or fibrocalcified plaques are more likely to have inadequate remodeling. Moreover, the arc of calcium was identified as an independent negative predictor of positive remodeling (Sabate et al., 1999). Calcified nodules, characterized by superficial large calcium, more frequently show negative remodeling than plaque rupture (Higuma et al., 2015). Lesions with calcified nodules seem to be more constrictive, as differentiated by the arc of calcification. The median maximum calcium arc in calcified nodules was 251°, whereas the median maximum calcium arc in ruptured calcific plaques was 56° (Higuma et al., 2015).

Instead of calculating the remodeling index, other imaging modalities, such as OCT, have attempted to evaluate vascular remodeling in other ways. Calcium deposits were considered as distorting if the luminal contour was narrowed or shifted by the shape of the underlying calcium (Ong et al., 2016). Then, the median number of calcium deposits distorting the lumen is greater in lesions in SAP patients than in those in patients with ST-segment-elevation myocardial infarction mediated by plaque rupture. The authors speculated that distorting calcifications occur when calcium deposits form without positive remodeling, resulting in luminal narrowing manifesting as SAP (Ong et al., 2016). However, whether this distortion is associated with vascular remodeling cannot be investigated, which suggests that the combined application of multiple imaging techniques is an inevitable trend in the future. The detailed plaque identification of OCT and good penetration of IVUS could be combined to better explore the impact of plaque composition and morphology on vascular remodeling.

## Conclusion

Vascular calcification is an important component in the atherosclerosis. Despite the uncertain association between atherosclerotic calcification and the prediction of future vascular events, there is an undoubted correlation between atherosclerotic calcification and plaque progression and stability. Calcification of differential amounts, sizes, shapes, and positions may play differential roles in plaque stability. Microcalcifications and spotty calcifications may represent an active stage of vascular calcification correlated with inflammation. The degree of calcification is inversely related to macrophage infiltration. Patients with larger calcifications are more often asymptomatic. Multiple and superficial calcifications are associated with plaque rupture and IPH, which might be due to the concentrated and asymmetrical distribution of biological stress in plaques. Calcified nodules, especially those protruding into the lumen, might result in the discontinuity of overlying collagen and endothelium with acute thrombosis. However, the impact of calcifications on plaque homeostasis depends not only on calcification characteristics, but also on the surrounding environment. The interactive effects of these important factors of calcifications and plaques still await further study.

## Author Contributions

XS and JG designed the review and the figures, planned the topic, reviewed the literature, and drafted the manuscript. QL and HC reviewed the literature and designed the table. FW reviewed the literature. RY designed the review and critically revised the manuscript. XL designed the review.

## Conflict of Interest

The authors declare that the research was conducted in the absence of any commercial or financial relationships that could be construed as a potential conflict of interest.
